# Dynamic changes in fecal microbiota in donkey foals during weaning: From pre-weaning to post-weaning

**DOI:** 10.3389/fmicb.2023.1105330

**Published:** 2023-01-27

**Authors:** Zhenwei Zhang, Bingjian Huang, Xu Gao, Xiaoyuan Shi, Xinrui Wang, Tianqi Wang, Yonghui Wang, Guiqin Liu, Changfa Wang

**Affiliations:** Liaocheng Research Institute of Donkey High-Efficiency Breeding and Ecological Feeding, Agricultural Science and Engineering School, Liaocheng University, Liaocheng, China

**Keywords:** weaning, fecal microbiota, bacteria, anaerobic fungi, archaea, donkey foal

## Abstract

**Introduction:**

A better understanding of the microbiota community in donkey foals during the weaning transition is a prerequisite to optimize gut function and improve feed efficiency. The objective of the present study was to investigate the dynamic changes in fecal microbiota in donkey foals from pre-to post-weaning period.

**Methods:**

A total of 27 fecal samples of donkey foals were collected in the rectum before morning feeding at pre-weaning (30 days of age, PreW group, *n* = 9), dur-weaning (100 days of age, DurW group, *n* = 9) and post-weaning (170 days of age, PostW group, *n* = 9) period. The 16S rRNA amplicon sequencing were employed to indicate the microbial changes during the weaning period.

**Results:**

In the present study, the cessation of breastfeeding gradually and weaning onto plant-based feeds increased the microbial diversity and richness, with a higher Shannon, Ace, Chao and Sobs index in DurW and PostW than in PreW (*p* < 0.05). The predominant bacterial phyla in donkey foal feces were Firmicutes (>50.5%) and Bacteroidota (>29.5%), and the predominant anaerobic fungi and archaea were Neocallimastigomycota and Euryarchaeota. The cellulolytic related bacteria including phylum Firmicutes, Spirochaetota and Fibrobacterota and genus norank_f_F082, *Treponema*, NK4A214_group, *Lachnospiraceae*_AC2044_group and *Streptococcus* were increased from pre-to post-weaning donkey foals (*p* < 0.05). Meanwhile, the functions related to the fatty acid biosynthesis, carbohydrate metabolism and amino acid biosynthesis were significantly enriched in the fecal microbiome in the DurW and PostW donkeys. Furthermore, the present study provided the first direct evidence that the initial colonization and establishment of anaerobic fungi and archaea in donkey foals began prior to weaning. The relative abundance of *Orpinomyces* were the highest in DurW donkey foals among the three groups (*p* < 0.01). In terms of archaea, the abundance of *Methanobrevibacter* were higher in PreW than in DurW and PostW (*p* < 0.01), but the abundance of *Methanocorpusculum* were significantly increased in DurW and PostW compared to PreW donkey foals (*p* < 0.01).

**Discussion:**

Altogether, the current study contributes to a comprehensive understanding of the development of the microbiota community in donkey foals from pre-to post-weaning period, which may eventually result in an improvement of the digestion and feed efficiency in donkeys.

## 1. Introduction

Donkey hindgut is vastly developed and consists of the cecum, colon and rectum (Julliand and Grimm, 2016). It’s a complex microbial ecosystem and is inhabited by highly dense and diverse microbiota, with bacteria, anaerobic fungi, methanogenic archaea, and ciliate protozoa involved (Edwards et al., 2020). Intestinal microbiota and their hosts have developed a complex and mutually adapted micro-ecological system (Santos et al., 2011). This steady microbiome-host balance is essential for the maintenance and ideal physiological function of intestine (Lindenberg et al., 2019).

Donkeys rely on the resident microbiota in hindgut to degrade dietary plant fiber to provide energy to support their growth and development (Dougal et al., 2012; Liu et al., 2020). A better understanding of the donkey hindgut microbiota is essential for the manipulation of the intestinal microbiota to optimize intestinal function and improve feed efficiency. In recent years, information is available in characterizing the hindgut microbiota in adult donkeys using high throughput sequencing of fecal samples (Liu et al., 2020; Li et al., 2022; Zhang et al., 2022a). As a suitable representation of the hindgut, feces are often used for microbial investigation in adult donkeys (Grimm et al., 2017). However, the fecal microbial population in donkey foal has never been studied during the weaning period. Weaning in donkey foals typically happens between 5 and 6 months of age in China, but the change from breast-feeding to plant-based feeds is gradual, as most donkey foals will have started ingesting plant-based solid feeds before weaning (Lindenberg et al., 2019).

From pre-weaning to post-weaning, donkey foals are subjected to a variety of stressors that include the separation from their dam, exposure to unfamiliar pens or novel pasture environments, and especially the switch from easily digestible milk to a more complicated adult-type feed (Zhang et al., 2022b). The cessation of milk and the subsequent shift in diet composition has a profound influence on the intestinal microbiota, as shown in other mammals, such as the calf (Luo et al., 2022), lamb (Li et al., 2018), deer Li et al., 2022), and piglet (Meng et al., 2020). Several studies have demonstrated that the development of the microbial ecosystem during weaning period may have an important impact on the microbial community structure in the adult and, consequently, the digestive efficiency and production performance (Zhang et al., 2017; Salem et al., 2018). The introduction of solid food leads to a new phase in the development of the microbiota, characterized by a significant increase in microbial abundance and diversity, and evolution toward a composition that is associated with adult individuals (Frese et al., 2015). Among the physiological and gastrointestinal factors influenced by the weaning transition, the microbiota alteration is likely to be considered as one of the keys leading to weaning stress (Weary et al., 2008). In addition, weaning stress usually causes intestinal microbiota related-disorders (Meng et al., 2020). Therefore, an in-depth understanding of foal gut microbiota during weaning transition is essential to optimize care of donkey foals and improve the health conditions.

However, to the very best of our knowledge, no work has reported on the dynamics of intestinal microbiota in donkey foals during weaning. An integrated investigation of the compositions and abundances of fecal bacteria, fungi, and archaea in donkey foals during weaning could be used to help develop microbial interventions to improve animal health and production performance. Therefore, the present study aimed at monitoring weaning-related changes in the microbial community structure in foal feces from pre-to post-weaning period using high-throughput MiSeq sequencing technique.

## 2. Materials and methods

### 2.1. Animals and sample collection

In the present study, 9 donkey foals (vaginal delivery) with the similar body weight and similar time of birth (from June 12, 2021 to July 4, 2021) were enrolled from the provincial Dezhou donkey original breeding farm. During the sampling period, donkey foals were clinically healthy and present no behavioral abnormalities. Following the practices commonly applied by Chinese equine breeders, donkey foal raising can be divided into three stages from pre-to post-weaning period.

From birth to day 50, donkey foals were maintained with their respective mares in the individual pens with straw bedding in a sandy paddock. Donkey foals had no access to concentrate and hay, but they received donkey milk and fresh water *ad libitum* during this pre-weaning period. From day 50 to day 150, donkey foals were still kept with their dams in a large sandy paddock and were not fed separately. In addition to the donkey milk, donkey foals had access to the mares feed bucket with wheat hay and supplemented concentrate *ad libitum* in the dur-weaning period. Donkey foals were abruptly weaned at 150 days of age. From day 150, foals were kept in one sand-bedded barn with wood shelters and were allowed access to wheat hay *ad libitum* and 1 kg/d concentrate diet (Hekangyuan Co., Ltd., Dezhou, Shandong Province, China). Donkey foals were fed twice daily at 08:00 and 18:00 in the post-weaning period. Water and a salt block were offered free access to all donkey foals.

Fecal samples of donkey foals were collected in the rectum using single-use gloves before morning feeding at pre-weaning (30 days of age), dur-weaning (100 days of age) and post-weaning (170 days of age) period. Each fecal sample was then put into sterile centrifuge tubes and kept in liquid nitrogen. All samples were transported to Liaocheng University laboratory immediately and stored at −80°C until DNA extraction. In addition, the body height (BH), body length (BL), thoracic girth (TG), thoracic depth (TD), thoracic width (TW), rump height (RH), rump length (RL), rump width (RW), and cannon bone circumference (CB) of donkey foals were determined before morning feeding by using a measuring stick or a tape.

### 2.2. The DNA extraction, PCR amplification, and 16S rRNA sequencing

Following the manufacturer’s protocol, microbial DNA of the fecal samples was extracted with the EZNA® Stool DNA Kit (Omega Bio-tek, Norcross, GA, United States). The extracted DNA purity and concentrations were determined using the NanoDrop 2000 UV–vis spectrophotometer (Thermo Scientific, Wilmington, United States).

The genomic DNA was then subjected to PCR amplification with the ABI GeneAmp® 9,700 PCR thermocycler (ABI, Foster City, CA, United States). For bacterial analysis, the V3-V4 hyper-variable region of the 16S rRNA gene was amplified with primers 338F (5′-ACTC CTAC GGGA GGCA GCAG-3′) and 806R (5′-GGAC TACH VGGG TWTC TAAT-3′) (Zhang et al., 2022a). The PCR amplification was performed in triplicate using the TransStart Fastpfu DNA polymerase in a volume of 20 μL containing 4 μL of 5 × FastPfu buffer, 2 μL of dNTPs (2.5 mM), 0.8 μL of forward and reverse primers (5 μM), 0.2 μL of BSA (bovine serum albumin), 0.4 μL of FastPfu polymerase, 10 ng of template DNA and ddH_2_O. For anaerobic fungi analysis, the 28S rRNA gene was amplified using primers GGNL1F (5′-CATA GAGG GTGA GAAT CCCG TA-3′) and GGNL4R (5′-TCAA CATC CTAA GCGT AGGT A-3′) (Nagler et al., 2019). For total archaea analysis, the 16S rDNA V4-V5 regions of the methanogen was amplified using primers 524F (5′-TGYC AGCC GCCG CGGT AA-3′) (Wei et al., 2013) and Arch958R (5′-YCCG GCGT TGAV TCCA ATT-3′) (Ma et al., 2018). The fungal and archaeal PCR amplifications were conducted with Pro Taq in a volume of 20 μL containing 10 μL of 2 × Pro Taq, 0.8 μL of forward and reverse primers (5 μM), 10 ng of template DNA and ddH_2_O. All PCR products were extracted from 2% agarose gel and further purified with DNA gel extraction kit (Axygen Bioscience, Union City, CA, United States) according to the manufacturer’s instructions and quantified by Quantus™ Fluorometer (Promega, United States).

Purified amplicons were ultimately pooled together in equimolar concentrations and high-throughput sequenced (paired-end, 2 × 300 bp) on an Illumina MiSeq platform (Illumina, San Diego, United States) at Majorbio Bio-Pharm Technology Co. Ltd (Shanghai, China) following standard protocols (Zhang et al., 2017).

### 2.3. Sequencing data processing and bioinformatic analysis

The raw data of sequencing reads were quality-filtered by Trimmomatic and merged by FLASH with the criteria as described by previous study (Zhang et al., 2017). To eliminate the influence of sequencing depth on microbial diversity and composition measurement, the number of reads from bacterial, fungal and archaeal sample was rarefied to 40,921, 30,469, and 34,214, respectively. Operational taxonomic units (OTUs) based on 97% identity were clustered with UPARSE (version 11.0),^1^ and chimeric sequences were removed. The taxonomy of specific OTU representative sequence (bacteria, fungi, and archaea) was analyzed by RDP Classifier (version 2.13)^2^ against the SILVA bacterial database (version 138) (Pruesse et al., 2007), UNITE fungi ITS database (version nt_v20210917) (Kõljalg et al., 2005), and SILVA archaea database (version 138/16S_archaea) through PYNAST (Caporaso et al., 2010) with the confidence threshold of 0.7 using QIIME.

Alpha diversity indices (Sobs, Chao, Shannon, and Simpson index) were measured by the MOTHUR (version 1.30.2). The Venn diagram and bar graphs were visualized by the R software (version 3.3.1). Beta diversity was determined by calculating the bray–curtis distance by QIIME (version 1.9.1) and showed by principal component analysis (PCA), principal coordinate analysis (PCoA) and non-metric multi-dimensional scaling (NMDS) plots. The microbial alterations among experimental groups were analyzed using partial least squares discriminant analysis (PLS-DA), and the results were plotted using R software (version 3.3.1). The Kruskal–Wallis H test and the One-way ANOVA was used to determine the phyla and genera that presented significant differences in abundance among groups using the scipy package in PYTHON and the stats package in R software (version 3.3.1). The linear discriminant analysis effect size (LEfSe) was used to determine statistically significant taxa in pre-, dur-, and post-weaning groups based on LDA scores >3.5 and *p* < 0.05. The co-abundant topological network of microbial genus was constructed using Networkx software. The PICRUSt (phylogenetic investigation of communities by reconstruction of unobserved states) transforms OTUs aligned against the SILVA, UNITE fungi and SILVA archaea database into the KEGG pathways (Kyoto Encyclopedia of Genes and Genomes).

All raw sequences after assembling and filtering were ultimately submitted to the NCBI Sequence Read Archive (SRA) with the accession number PRJNA903673.

### 2.4. Statistical analysis

Body measurements of donkey foals were conducted using PROC MEANS procedures in SAS 9.4 software (Statistical Analysis for Windows, SAS Institute Inc., Cary, NC, United States), and the data were expressed as Mean ± SD. Significant differences of alpha diversity index, microbial abundance at phylum- and genus-level, enriched KEGG Module pathways and COG functions among PreW, DurW, and PostW groups were determined using Wilcoxon rank-sum test in R software (version 4.0.3). The presented data are expressed as least-squares means (SEM) with their standard errors. Statistical significance was set at *p* < 0.05 and a trend was declared at *p* < 0.1. The correlations between donkey fecal microbiota and donkey body measurements were evaluated *via* Pearson’s correlation analysis using the pheatmap package (version 3.3.1) in R.

## 3. Results

### 3.1. Body measurements of donkey foals

As shown in Table 1, with the age increasing, the body measurements including BH, BL, TG, TD, TW, RH, RL, RW, and CB linearly increased from pre-weaning to post-weaning period.

**Table 1:** Descriptive values for donkey foals are shown related to body measurement.

Items	BH	BL	TG	TD	TW	RH	RL	RW	CB
PreW	89 ± 1.3^c^	60 ± 1.4^c^	73 ± 1.8^c^	33 ± 0.6^b^	17 ± 0.5^c^	90 ± 1.5^c^	24 ± 0.6^c^	21 ± 0.8^c^	10 ± 0.2^c^
DurW	102 ± 1.1^b^	90 ± 2.2^b^	94 ± 1.9^b^	33 ± 0.8^b^	22.1 ± 0.8^b^	104 ± 1.1^b^	30 ± 0.4^b^	25 ± 0.6^b^	13.2 ± 0.3^b^
PostW	117 ± 3.1^a^	117 ± 2.7^a^	119 ± 3.2^a^	44 ± 1.2^a^	28 ± 0.9^a^	121 ± 2.8^a^	37 ± 1.1^a^	32 ± 1.0^a^	14 ± 0.3^a^

^a,b,c^Different superscript letters within a row represents values with significantly different at *P* < 0.05. PreW, pre-weaning donkeys; DurW, during weaning donkeys; PostW, post-weaning donkeys; BH, body height (cm); BL, body length (cm); TG, thoracic girth (cm); TD, thoracic depth (cm); TW, thoracic width (cm); RH, rump height (cm); RL, rump length (cm); RW, rump width (cm); CB, circumference of cannon bone (cm); data expressed as Mean ± SEM.

### 3.2. Fecal bacteria changes in donkey foals from pre- to post-weaning period

The Shannon, Ace, Chao and Sobs index of PreW group was lower than that of DurW and PostW group (Table 2, *p* < 0.01), which indicated that the PreW group had lower bacterial diversity and richness in comparison with DurW and PostW groups.

**Table 2:** Alpha diversity indices of bacteria, anaerobic fungi, and archaea among different groups.

Items		PreW	DurW	PostW	SEM	*P*-value
Bacteria	Shannon	3.1^b^	5.9^a^	5.6^a^	0.23	<0.01
	Ace	453.2^b^	1840.6^a^	1677.7^a^	82.9	<0.01
	Chao	453.7^b^	1853.3^a^	1686.3^a^	86.1	<0.01
	Sobs	343.4^b^	1521.1^a^	1375.2^a^	69.6	<0.01
Anaerobic fungi	Shannon	1.0	1.0	1.0	0.09	0.97
	Ace	6.8^b^	30.9^ab^	18.0^ab^	5.05	0.02
	Chao	7.5^b^	29.2^a^	17.6^ab^	4.86	0.03
	Sobs	7.5^b^	27.0^a^	14.9^ab^	4.68	0.04
Archaea	Shannon	0.9^b^	1.3^a^	1.2^ab^	0.11	0.06
	Ace	23.4^b^	26.6^b^	46.1^a^	5.96	0.03
	Chao	22.8^b^	24.4^b^	32.7^a^	2.79	0.04
	Sobs	22.3	22.8	26.9	2.71	0.44

^a,b^Different superscript letters within a row represents values with significantly different at *P* < 0.05. PreW, pre-weaning donkeys; DurW, during weaning donkeys; PostW, post-weaning donkeys; SEM, standard error of the mean.

**Figure 1:**
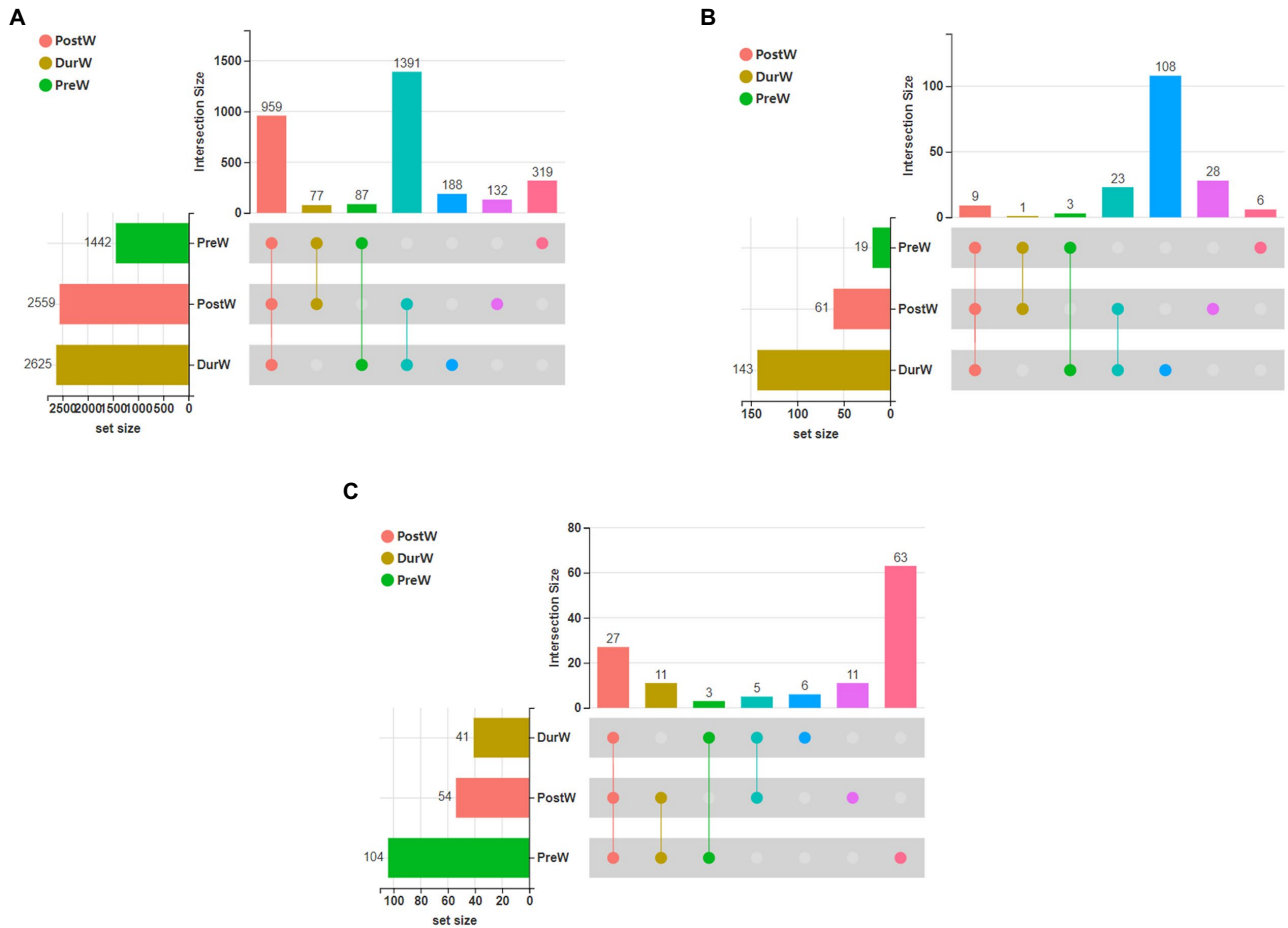
The UpSet Venn diagram presenting the distribution of microbial bacterial **(A)**, anaerobic fungi **(B)**, and archaeal **(C)** community OTUs among different groups. PreW, pre-weaning donkeys; DurW, during weaning donkeys; PostW, post-weaning donkeys.

The UpSet Venn diagram (Figure 1A) showed the distribution of bacterial community OTUs. There were 1,442, 2,559 and 2,625 OTUs observed in the PreW, DurW and PostW groups, respectively. The PreW donkey foals shared a bacterial community, including 959 OTUs, with DurW and PostW.

**Figure 2:**
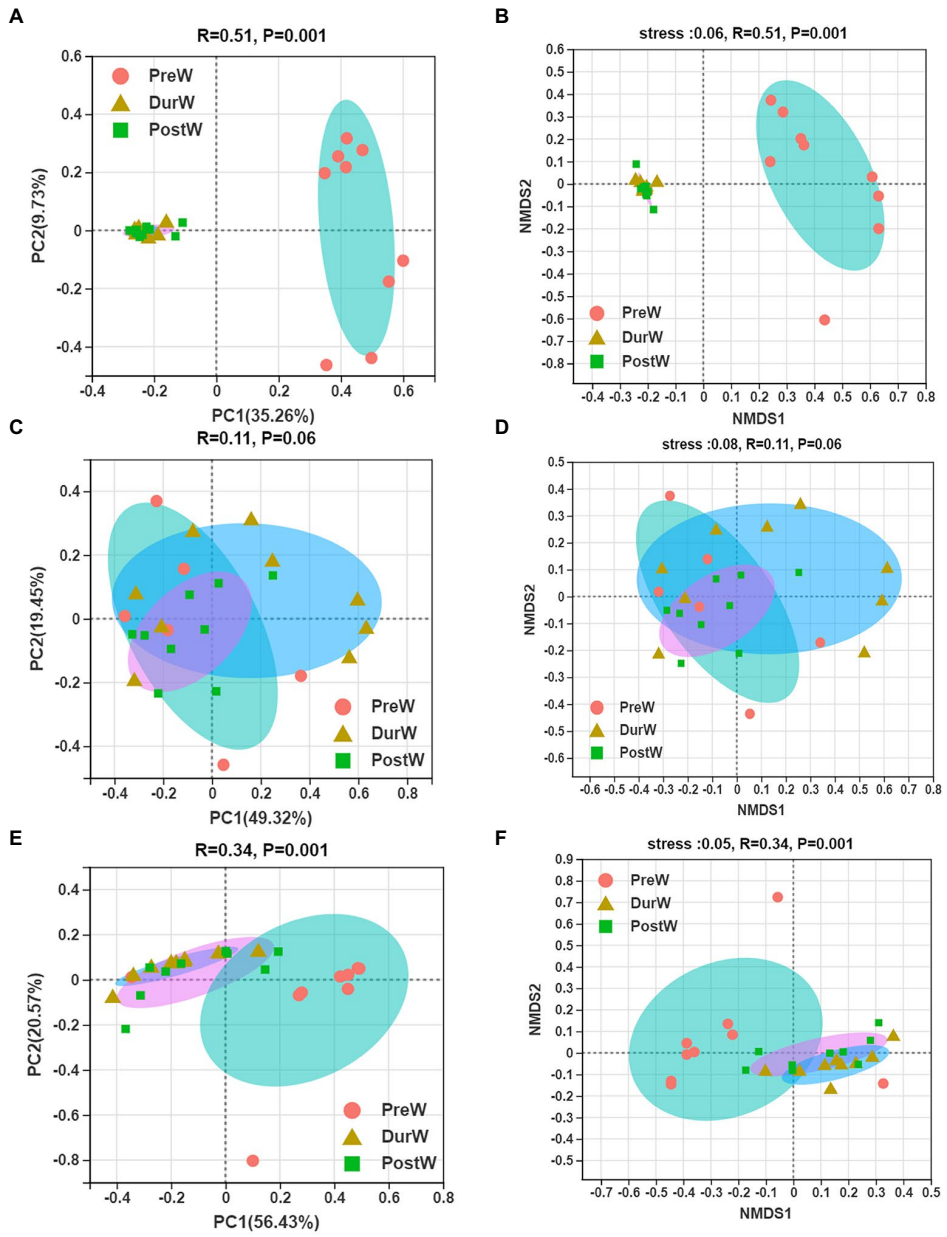
Principal co-ordinates analysis (PCoA) and Nonmetric multidimensional scaling analysis (NMDS) of microbial community composition among different groups. (**A)** The PCoA analysis of bacteria. (**B)** The NMDS analysis of bacteria. (**C)** The PCoA analysis of anaerobic fungi. (**D)** The NMDS analysis of anaerobic fungi. (**E)** The PCoA analysis of archaea. (**F)** The NMDS analysis of archaea; PreW, pre-weaning donkeys; DurW, during weaning donkeys; PostW, post-weaning donkeys.

The results of PCoA and NMDS for bacteria with weighted uniFrac distances showed that the PreW group were obviously separated from DurW and PostW groups at the OTU level (Figures 2A,B, ANOSIM: *R* = 0.51, *p* = 0.001).

**Figure 3:**
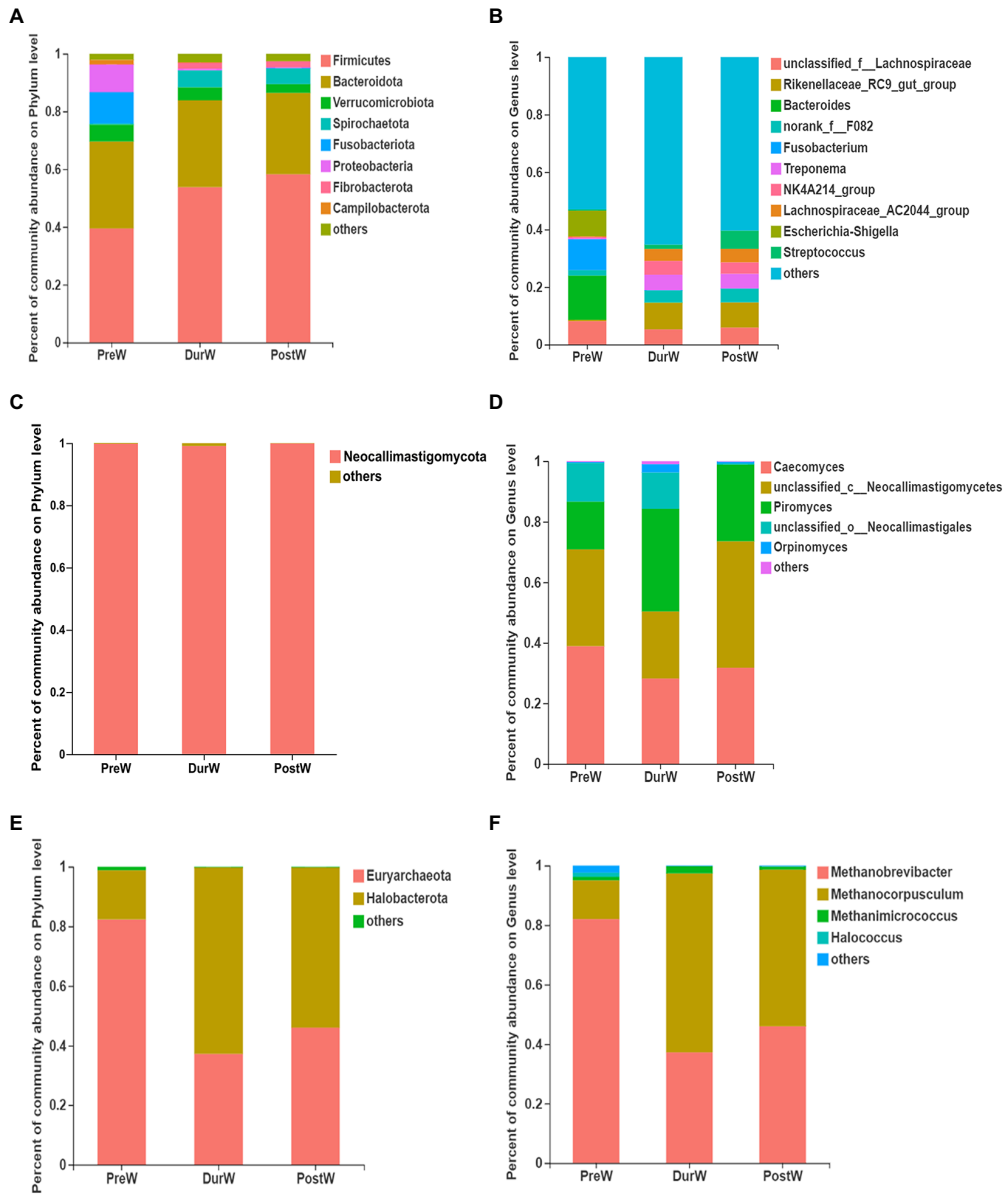
Composition of the predominant microbiota among different groups (accounting for ≥0.01% of the total sequences in at least one samples). (**A)** The predominant bacteria at phylum level. (**B)** The predominant bacteria at genus level. (**C)** The predominant anaerobic fungi at phylum level. (**D)** The predominant anaerobic fungi at genus level. (**E)** The predominant archaea at phylum level. (**F)** The predominant archaea at genus level; PreW, pre-weaning donkeys; DurW, during weaning donkeys; PostW, post-weaning donkeys.

**Figure 4:**
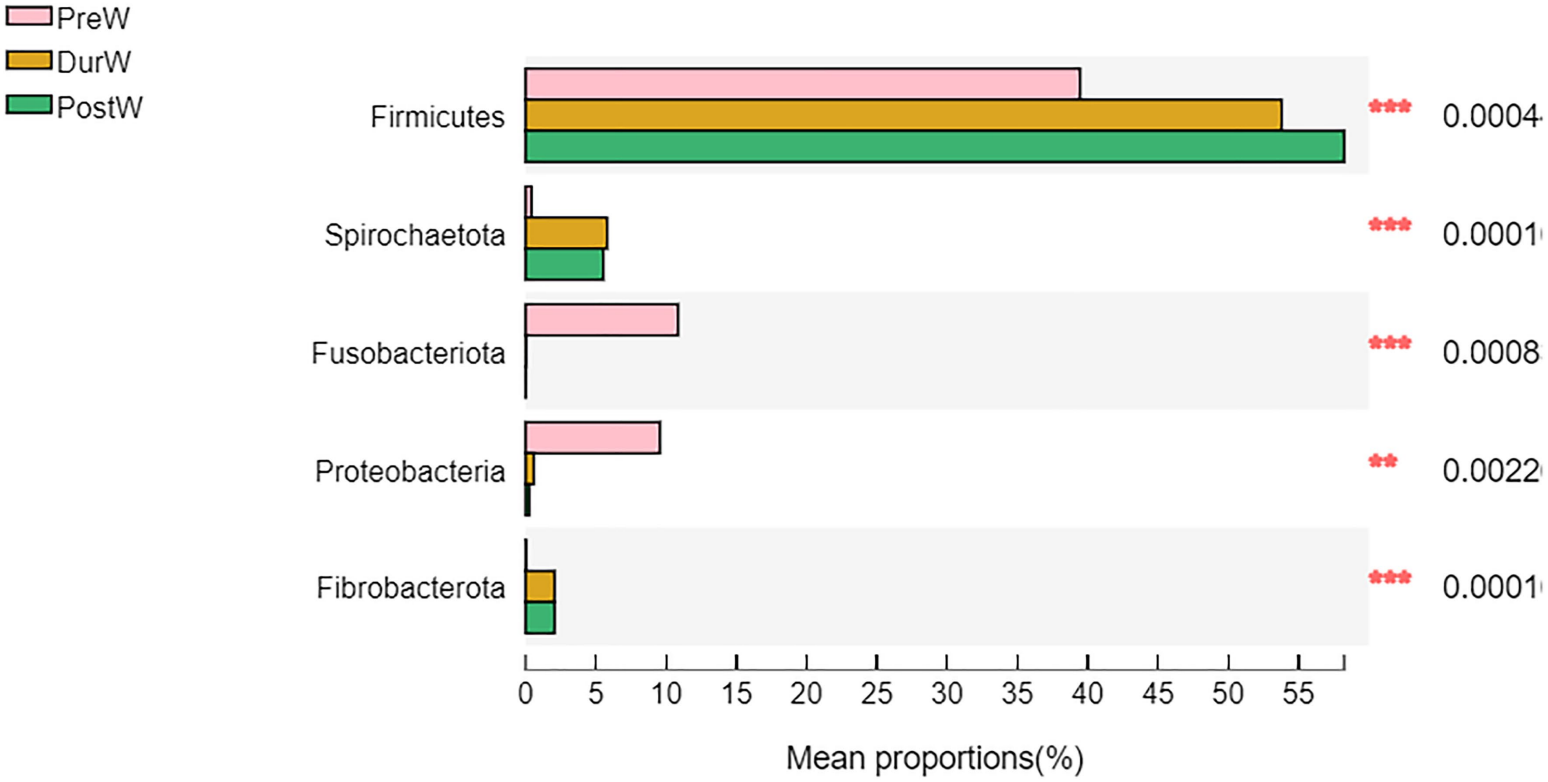
Difference of the predominant microbiota at phylum level among different groups (abundance of the microbiota is expressed as a percentage). PreW, pre-weaning donkeys; DurW, during weaning donkeys; PostW, post-weaning donkeys; **p < 0.05; ***p < 0.01.

The relative abundance of bacterial phyla accounting for ≥0.01% of the total sequences in at least one samples were presented in Figure 3A. Among these phyla, Firmicutes and Bacteroidota had relatively high abundance, at mean abundance levels of 50.5 and 29.5%, respectively (Figure 3A). The relative abundance of Firmicutes, Spirochaetota and Fibrobacterota in PreW group were lower than that in DurW and PostW groups (Figure 4, *p* < 0.01). However, the relative abundance of Fusobacteriota and Proteobacteria in PreW donkey foals were higher in comparison with DurW and PostW donkey foals (*p* < 0.01).

**Figure 5:**
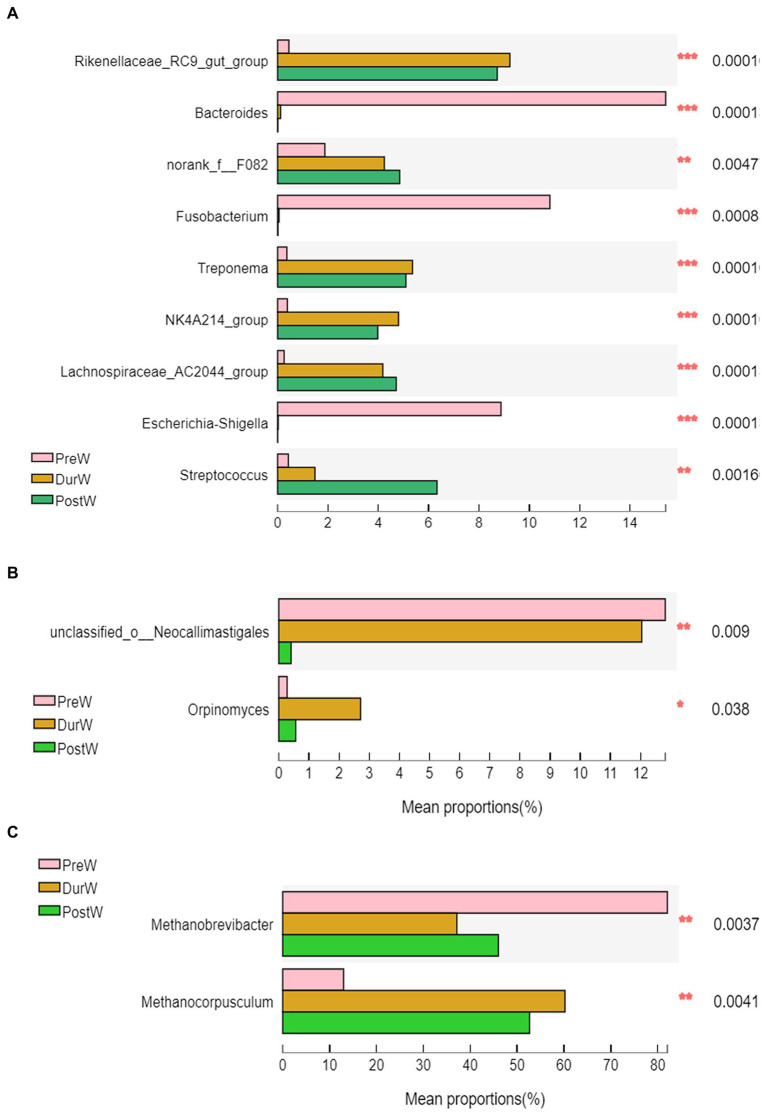
Difference of the predominant microbiota at genus level among different groups (abundance of the microbiota is expressed as a percentage). (**A)** The different bacteria. (**B)** The different anaerobic fungi. (**C)** The different archaea; PreW, pre-weaning donkeys; DurW, during weaning donkeys; PostW, post-weaning donkeys; *p < 0.05; **p < 0.01, ***p<0.001.

The top 10 abundant bacterial genera of fecal microbiota in pre-, dur- and post-weaning donkey foals were unclassified_f_*Lachnospiraceae*, *Rikenellaceae*_RC9_gut_group, *Bacteroides*, norank_f_F082, *Fusobacterium*, *Treponema*, NK4A214_group, *Lachnospiraceae*_AC2044_group, *Escherichia*-*Shigella* and *Streptococcus* (Figure 3B). The relative abundance of *Rikenellaceae*_RC9_gut_group, norank_f_F082, *Treponema*, NK4A214_group, *Lachnospiraceae*_AC2044_group and *Streptococcus* in PreW donkey foals were significantly lower than that in DurW and PostW donkey foals (Figure 5A, *p* < 0.01). In contrast, the relative abundance of *Fusobacterium* and *Escherichia*-*Shigella* in PreW group were obviously higher than in DurW and PostW groups (*p* < 0.01).

### 3.3. Fecal anaerobic fungi changes in donkey foals from pre- to post-weaning period

Compared to the PreW group, both the DurW and PostW group had the higher Ace, Chao and Sobs index (Table 2, *p* < 0.05). However, there was no significant difference among PreW, DurW and PostW groups for Shannon index (*p* > 0.05).

The distribution of anaerobic fungi community OTUs were presented in Figure 1B. There were 19, 61 and 143 OTUs in the PreW, DurW, and PostW donkey foals, respectively. The PreW donkey foals shared an anaerobic fungi community, including 9 OTUs, with DurW and PostW.

The PCoA and NMDS based on unweighted UniFrac distance revealed that the fecal anaerobic fungi of donkey foals showed no obvious segregation from pre-weaning to post-weaning (Figures 2C,D, ANOSIM: *R* = 0.11, *p* = 0.06).

The anaerobic fungi sequences determined at the phylum level largely belonged to Neocallimastigomycota (>99.9%), and there was no significant difference among PreW, DurW, and PostW groups (Figure 3C, *p* > 0.05). At genus level, *Caecomyces*, unclassified_c__*Neocallimastigomycetes*, *Piromyces*, unclassified_o__*Neocallimastigales* and *Orpinomyces* were the predominant genera in all samples (Figure 3D). The relative abundance of unclassified_o__*Neocallimastigales* in PreW and DurW donkey foals were significantly higher than in PostW donkey foals (Figure 5B, *p* < 0.01). The relative abundance of *Orpinomyces* were the highest in DurW donkey foals among the three groups (Figure 5B, *p* < 0.01), but there was no significant difference between PreW and PostW donkey foals (*p* > 0.05).

### 3.4. Fecal archaea changes in donkey foals from pre- to post-weaning period

As shown in Table 2, the PreW group had lower Shannon index than that in the DurW and PostW group (*p* < 0.05). In addition, the Ace and Chao index in PreW group was also lower than that in the DurW and PostW group (*p* < 0.05).

There were 19, 61 and 143 archaeal community OTUs in the PreW, DurW, and PostW groups (Figure 1C). A total of 27 OTUs could be observed in all three groups and there were 6, 11, and 63 unique OTUs in the PreW, DurW, and PostW donkey foals, respectively.

In terms of beta diversity of archaea community at the OTU level in donkey foals, unweighted UniFrac PCoA and NMDS showed that the separation of PreW group from DurW and PostW occurred (Figures 2E,F, ANOSIM: *R* = 0.34, *p* = 0.001), but no obvious separation of the samples between DurW and PostW.

The sequences detected at archaeal phylum level mainly belonged to Euryarchaeota and Halobacterota (Figure 3E). The predominant genera detected in the present study were *Methanobrevibacter*, *Methanocorpusculum*, *Methanimicrococcus* and *Halococcus* (Figure 3F). As shown in Figure 5C, the relative abundance of *Methanobrevibacter* were significantly higher in PreW donkey foals than in DurW and PostW donkey foals (*p* < 0.01), however, the relative abundance of *Methanocorpusculum* in PreW group was remarkably lower than in DurW and PostW groups (*p* < 0.01).

### 3.5. Microbial biomarkers of different groups

**Figure 6:**
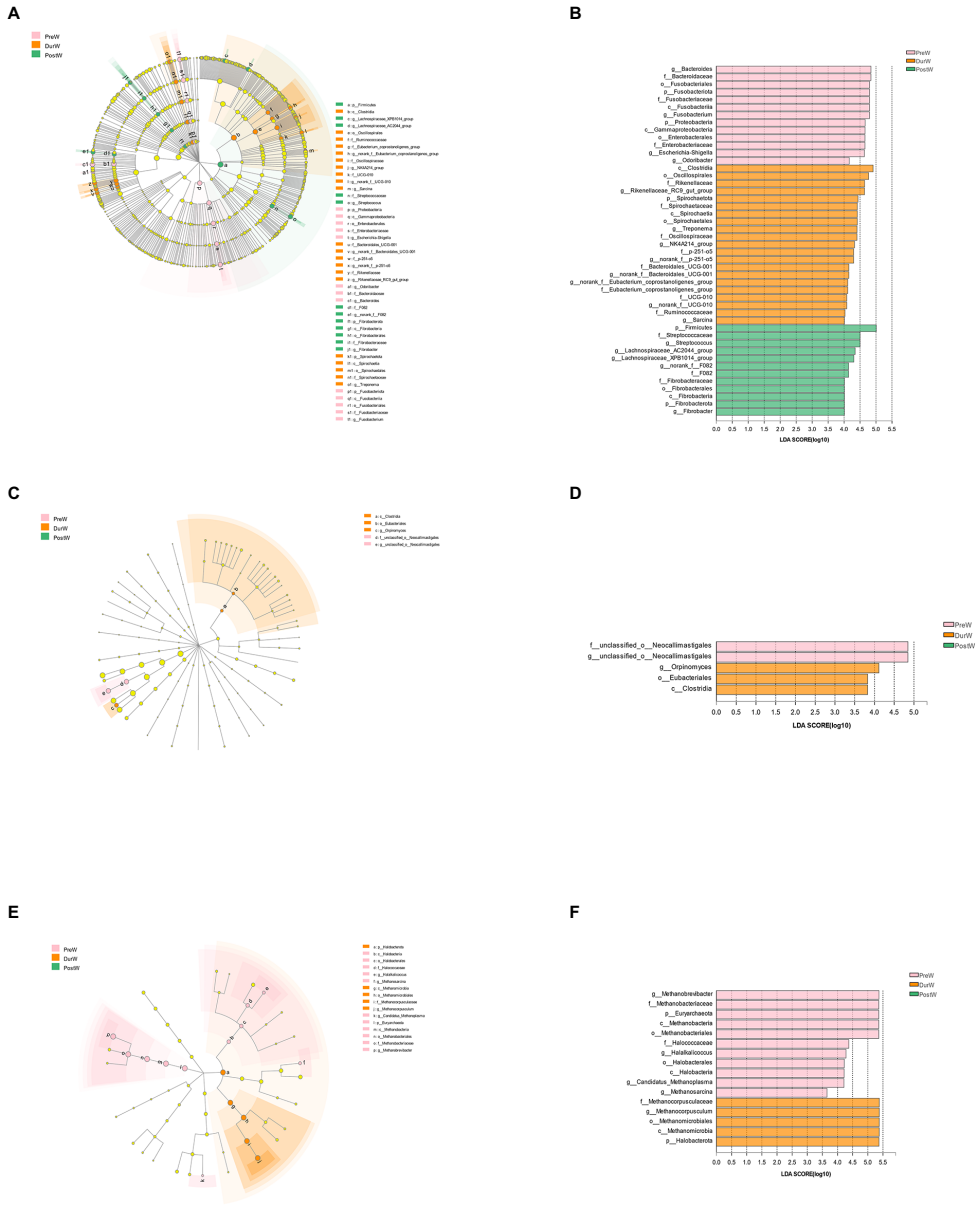
The LEfSe analysis showed the biomarkers of the microbial community in donkey foals during weaning period. The cladogram indicating the differences in relative abundance of taxa among PreW, DurW, and PostW groups; and the bar column shows the microbial taxa with significant differences among three groups (LDA score > 3). The length of the bar column represents the LDA score. **(A)** The cladogram of bacteria. **(B)** The LDA plot of bacteria. **(C)** The cladogram of anaerobic fungi. **(D)** The LDA plot of anaerobic fungi. **(E)** The cladogram of archaea. **(F)** The LDA plot of archaea; PreW, pre-weaning donkeys; DurW, during weaning donkeys; PostW, post-weaning donkeys.

To identify the differences in fecal microbiota of donkey foals among PreW, DurW and PostW groups, linear discriminant analysis effect size (LEfSe) analysis was performed from phylum to genus level (Figure 6).

The bacteria with significant differences in the PreW group were genus g_*Bacteroides*, g_*Fusobacterium*, g_*Escherichia-Shigella* and g_*Odoribacter*, the bacterial biomarkers for DurW group were genus g_*Rikenellaceae*_RC9_gut_group, g_*Treponema*, g_NK4A214_group, g_norank_f_p-251-o5, g_norank_f_*Bacteroidales*_UCG-001, g_norank_f_UCG-010 and g_*Sarcina*; the bacterial biomarkers for PostW group were genus g_*Streptococcus*, g_*Lachnospiraceae*_AC2044_group, g_*Lachnospiraceae*_XPB1014_group, g_norank_f_F082 and g_*Fibrobacter* (Figures 6A,B).

For anaerobic fungi, only 5 biomarkers with statistical differences between PreW and DurW were determined using LEfSe (2 in the PreW, 3 in the DurW), as shown in Figures 6C,D. Unclassified_o_*Neocallimastigales* and g_unclassified_o_*Neocallimastigales* was significantly enriched in the PreW group, and the DurW group was significantly enriched in genus g_*Orpinomyces*.

A total of 16 biomarkers of archaea with statistical differences between PreW and DurW were observed by LEfSe analysis (11 in the PreW, 5 in the DurW; Figures 5E,F). The dominant archae from the fecal samples in the PreW group were mainly from the genus g_*Methanobrevibacter*, g_*Halalkalicoccus*, g_*Candidatus*_*Methanoplasma* and g_*Methanosarcina*, while the archaeal biomarkers for DurW group were genus g_*Methanocorpusculum* and class c_*Methanomicrobia*.

### 3.6. The co-occurrence network analysis on microbiota

**Figure 7:**
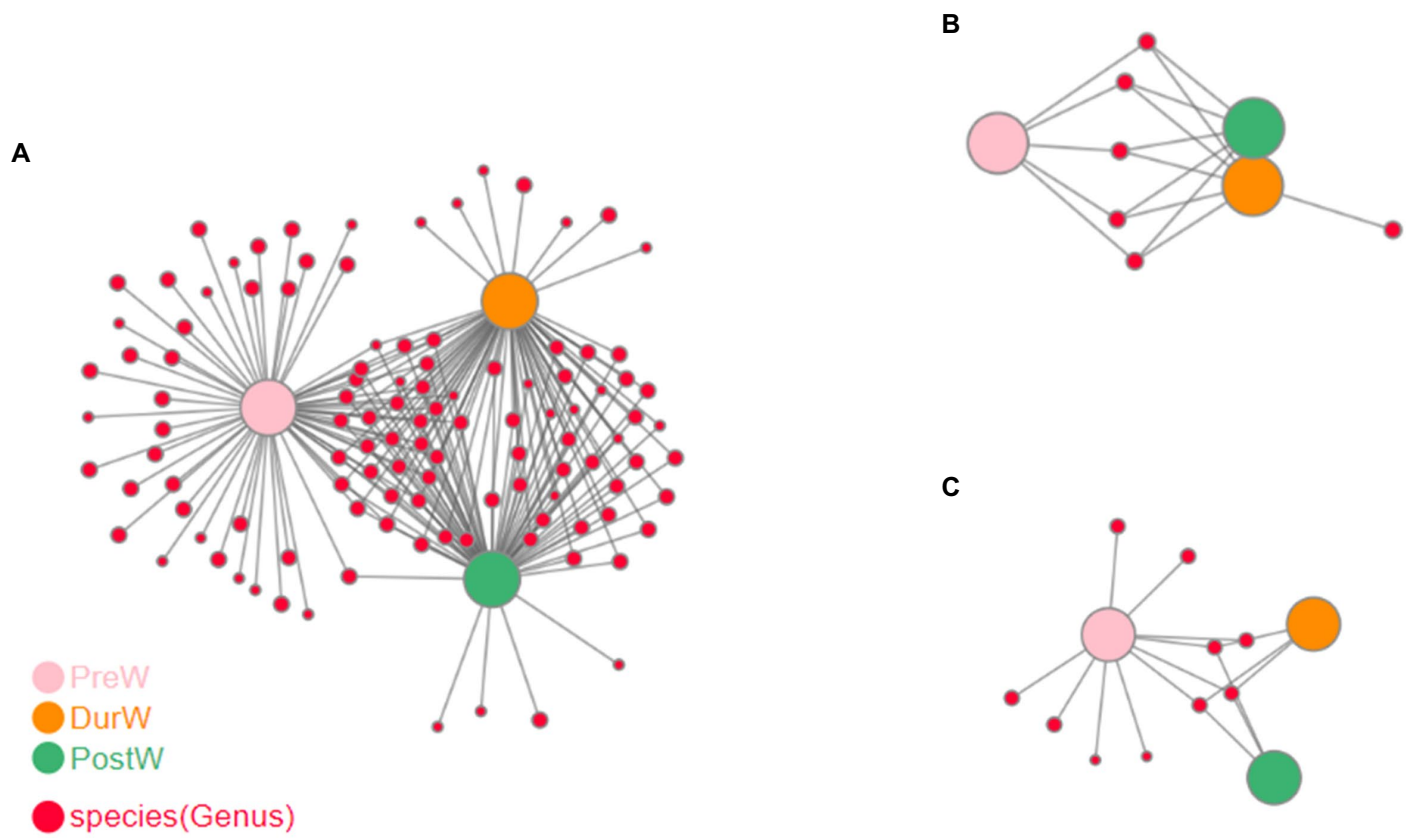
Co-occurrence network analysis on microbiota among different groups. (**A)** The network analysis of bacteria. (**B)** The network analysis of anaerobic fungi. (**C)** The network analysis of archaea; PreW, pre-weaning donkeys; DurW, during weaning donkeys; PostW, post-weaning donkeys.

The co-occurrence network analysis highlighted the differences among different weaning periods. There were more strongly bacterial and archaeal networks of DurW-PostW than the network of PreW-DurW and PreW-PostW (Figures 7A,C). The anaerobic fungi networks of DurW-PostW were weaker, and there were similarly networks of PreW-DurW and PreW-PostW (Figure 7B).

### 3.7. Correlation of differentially donkey fecal microbiota and body measurements

**Figure 8:**
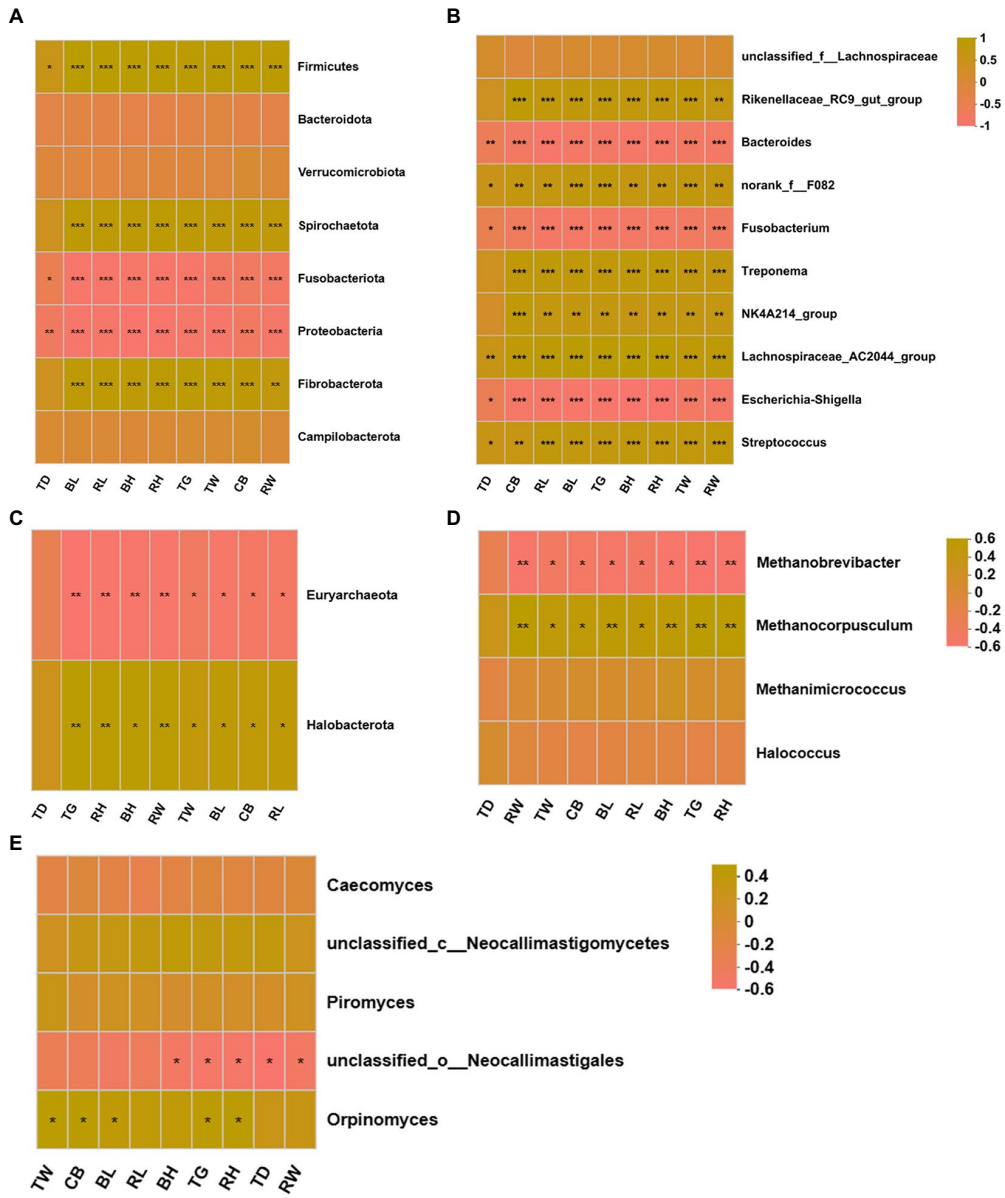
Correlation heatmap of differentially donkey fecal microbiota and body measurements. Each row in the graph represents a microbiota phylum/genus, each column represents a body measurement, the color in the graph indicates the Pearson coefficient between the microbial phylum/genus and donkey body measurements, and the brick yellow indicates positive correlation. The pale pink is representative negative correlation. The darker color indicate the greater the correlation. **(A)** Between donkey body measurements and bacteria at phylum level. **(B)** Between donkey body measurements and bacteria at genus level. **(C)** Between donkey body measurements and anaerobic fungi at phylum level. **(D)** Between donkey body measurements and anaerobic fungi at genus level. **(E)** Between donkey body measurements and archaea at genus level. *p < 0.05; **p<0.01; ***p<0.001. BH, body height; BL, body length; TG, thoracic girth; TD, thoracic depth; TW, thoracic width; RH, rump height; RL, rump length; RW, rump width; CB, circumference of cannon bone.

The possible correlations between the differentially donkey fecal microbiota and body measurements were determined based on Spearman’s correlation (Figure 8). At phylum level, the bacterial Firmicutes, Spirochaetota, and Fibrobacterota were positively correlated with donkey BH, BL, TG, TW, RH, RL, RW, and CB (Figure 8A, *p* < 0.05), but the bacterial Fusobacteriota and Proteobacteria was negatively related with donkey body measurements (*p* < 0.05). At genus level, the bacterial *Rikenellaceae*_RC9_gut_group, norank_f_F082, *Treponema*, NK4A214_group, *Lachnospiraceae*_AC2044_group and *Streptococcus* were positively correlated with donkey body measurements (Figure 8B, *p* < 0.05). Conversely, the donkey body measurements were negatively correlated with the bacterial *Bacteroides*, *Fusobacterium* and *Escherichia*-*Shigella* (*p* < 0.05). For fecal archaea, there was a significantly positive correlation between donkey body measurements and phylum Halobacterota (Figure 8C, *p* < 0.05), but donkey body measurements were negatively correlated with Euryarchaeota (*p* < 0.05). At genus level of archaea, the *Methanocorpusculum* were positively corelated with BH, BL, TG, TW, RH, RL, RW, and CB, however, the donkey body measurements were negatively corelated with *Methanobrevibacter* (Figure 8D, *p* < 0.05). For anaerobic fungi, no obvious correlation was observed between donkey body measurements and Neocallimastigomycota (*p* > 0.05). But at genus level, there was a positive correlation between *Orpinomyces* and TW, CB, BL, TG and RH (Figure 8E, *p* < 0.05). In addition, there was a negative correlation between unclassified_o__*Neocallimastigales* and donkey body measurements including BH, TG, RH, TD and RW (*p* < 0.05).

### 3.8. Differences of microbial function in donkey foals from pre- to post-weaning period

**Figure 9:**
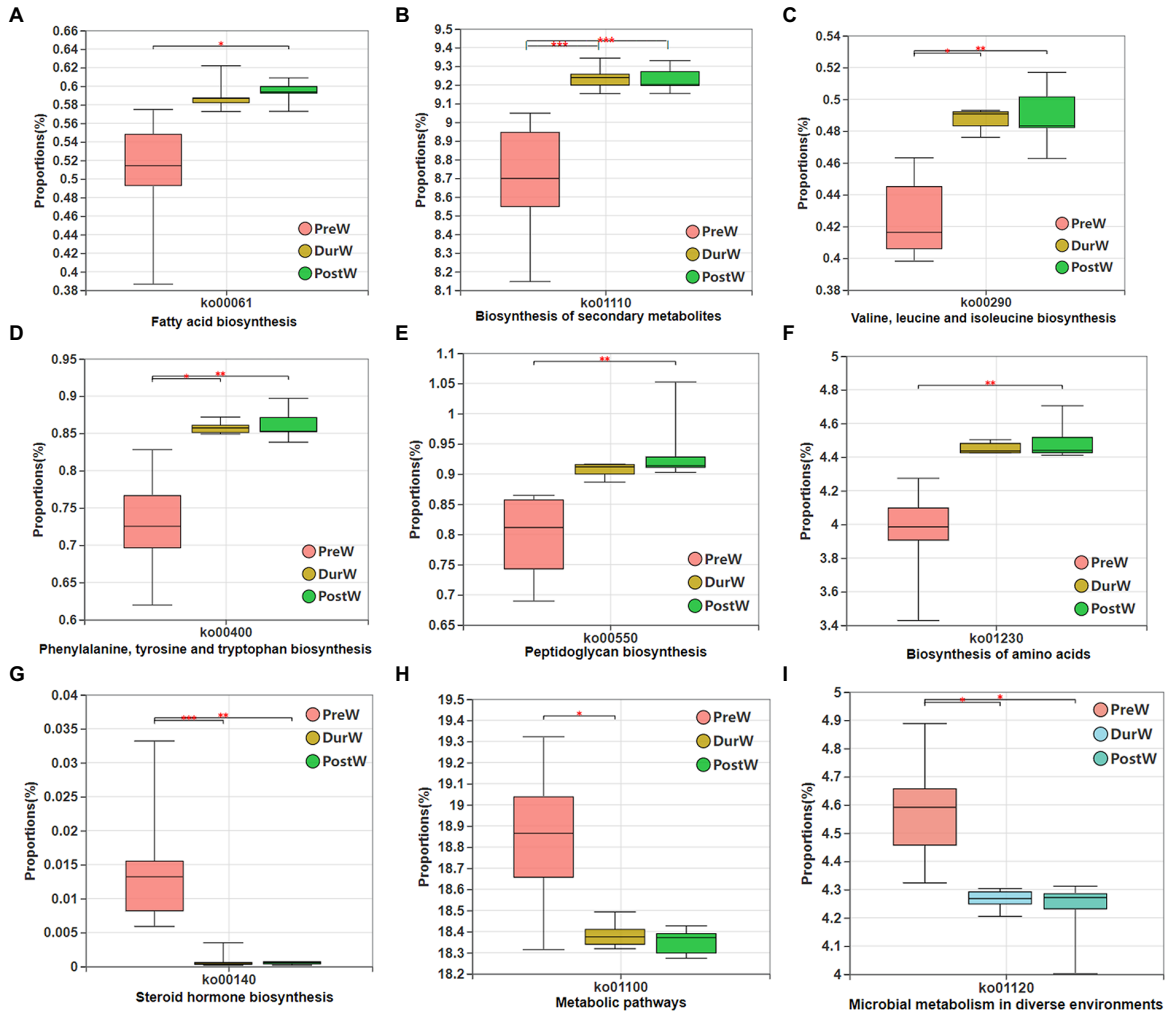
Variations in composition of bacterial KEGG metabolic pathways inferred by PICRUSt. KEGG, Kyoto Encyclopedia of Genes and Genomes; PICRUSt, the phylogenetic investigation of communities by reconstruction of unobserved states; PreW, pre-weaning donkeys; DurW, during weaning donkeys; PostW, post-weaning donkeys. *p < 0.05; **p<0.01; ***p<0.001.

The potential functional capacity of fecal bacteria of donkey foals were inferred by PICRUSt using the16S rRNA data. A total of 178 differential KEGG metabolic pathways (level 3) were detected, and 9 differential KEGG metabolic pathways among PreW, DurW, and PostW groups are presented in Figure 9. The relative abundance of pathways including fatty acid biosynthesis (Figure 9A), biosynthesis of secondary metabolites (Figure 9B), Valine, leucine, and isoleucine biosynthesis (Figure 9C), phenylalanine tyrosine and tryptophan biosynthesis (Figure 9D), peptidoglycan biosynthesis (Figure 9E) and biosynthesis of amino acids (Figure 9F) in PreW donkey foals were significantly lower than that in DurW and PostW donkey foals (*p* < 0.05). Conversely, the relative abundance of pathways including steroid hormone biosynthesis (Figure 9G), metabolic pathways (Figure 9H) and microbial metabolism in diverse environments (Figure 9I) in PreW group were remarkably higher than in DurW and PostW groups (*p* < 0.05).

**Figure 10:**
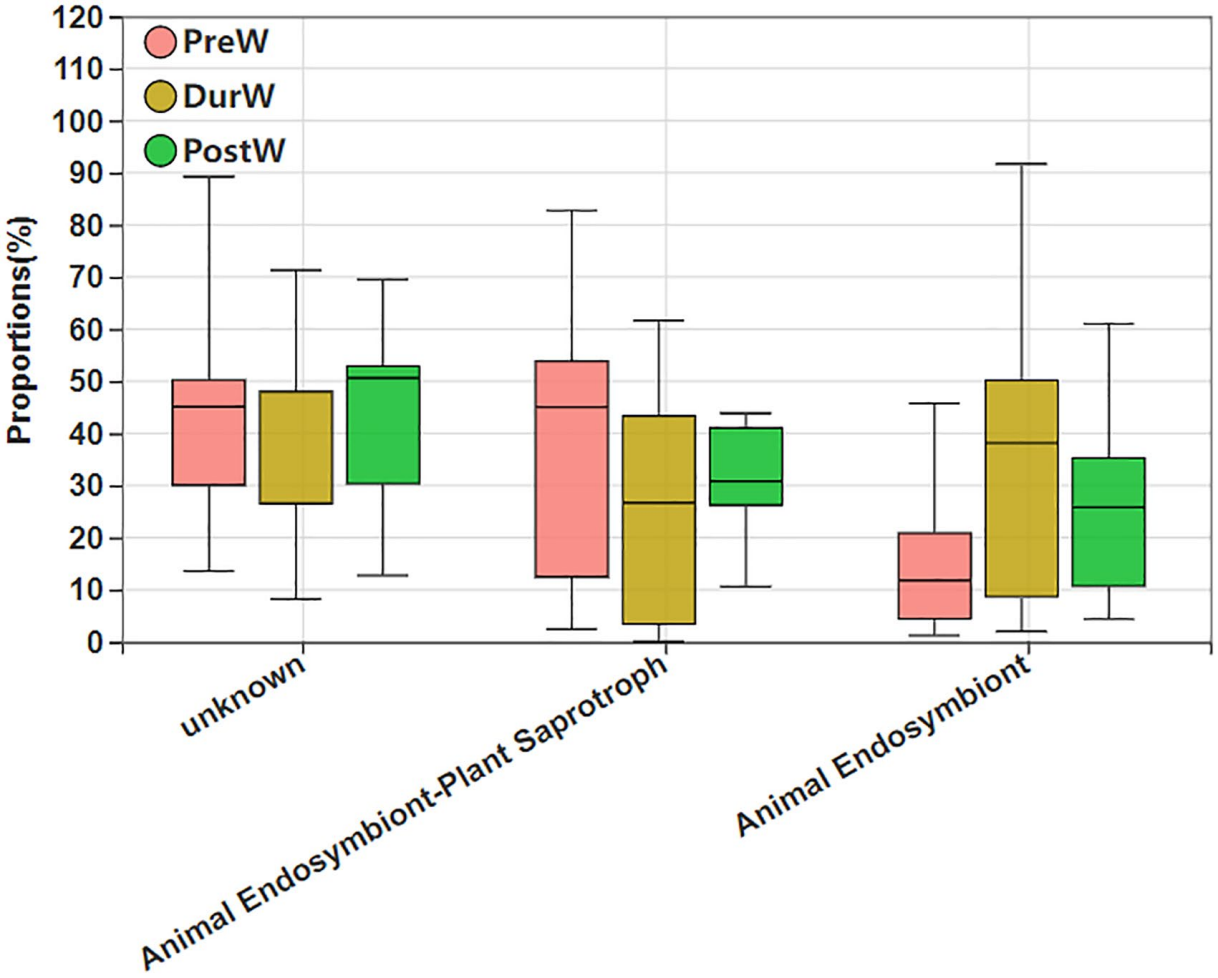
Variations in composition of anaerobic fungi functional groups inferred by FUNGuild. FUNGuild, fungi functional guild; PreW, pre-weaning donkeys; DurW, during weaning donkeys; PostW, post-weaning donkeys.

The potential functional abundance of fecal anaerobic fungi of donkey foals were characterized by FUNGuild using the 28S rRNA data (Figure 10). There was no significant difference among PreW, DurW, and PostW groups for anaerobic fungi functional abundance (*p* > 0.05).

## 4. Discussion

Weaning is one of the most important events in the early life of donkey foals. The large-scale donkey farms in China often use gradual weaning, allowing donkey foals to suckle the colostrum and then slowly reducing the amount of the breastfeed. From pre-weaning to post-weaning period, the gut microbiota composition of donkey foals was not immutable, but rather a dynamic alteration process (Lindenberg et al., 2019). During the weaning period, the diet of donkey foals gradually changes from a high-fat, low-fiber milk to a plant-based solid feed. The diet alteration was usually considered to be one of the important driving forces in the development and changes of gut microbiota in animals (Guevarra et al., 2018). Moreover, the process of microbial establishment in the gastrointestinal tract of donkey foals plays an important role for host health and nutrition (Lindenberg et al., 2019). However, few studies have focused on the development of the gut microbiome in donkey foals during the weaning period. To our best of knowledge, the present study is novel in being the first study to investigate the fecal microbiota (including bacteria, anaerobic fungi, and archaea) of donkey foals over a whole weaning period.

Microbial diversity and richness are usually applied to evaluate the stability of gut ecosystem in animals. Konopka (2009) noted that the high microbial diversity and richness could help to maintain the stability and resistance of ecosystem under environmental pressure. The high microbial diversity and richness is regarded as a sign of matured gut microbiota and it is also believed beneficial for host health (Turnbaugh et al., 2009; Li et al., 2020). In terms of present diversity and richness of donkey foal feces, the microbial Shannon, Ace, Chao and Sobs index in donkey foals were all increased with the changes in donkey foals’ breast milk and starter intake from pre-to post-weaning period. This result indicated that the gut microbiota of donkey foals may be established more completely after weaning to better adapt to the digestion of plant-based feed. In addition, the bacterial, anaerobic fungi and archaeal complexity before weaning was lower than the post-weaning stage, which was consistent with previous studies using other animals like swine, horse, and lamb (Kim et al., 2012; Mach et al., 2017; Wang et al., 2022).

Regarding the beta diversity, the PcoA and NMDS analysis showed that there were obvious differences in donkey foal fecal microbiota composition from pre-weaning to post-weaning period. However, no significant difference occurred between dur-weaning and post-weaning donkey foals. The cessation of breastfeeding may result in the microbial community changes due to the decreasing influence of specific milk components on the bacteria, anaerobic fungi and archaea (Lindenberg et al., 2019). As donkey foals at weaning have had a subtle intake of plant-based feed for at least several months, a microbial composition associated with this diet is to be expected.

### 4.1. Fecal bacteria changes in donkey foals

Consistent with the previous reports on donkeys (Zhang et al., 2022a), Firmicutes and Bacteroidetes were the most predominant bacteria (represented >80% of all sequences). From pre-weaning to post weaning, the proportion of Firmicutes, Spirochaetota and Fibrobacterota increased, whereas the proportion of Fusobacteriota and Proteobacteri decreased. Both Proteobacteria and Fusobacteria have been found in the feces of newborn animals prior weaning, which act as stains to promote the development of the gut microbiome in the subsequent growth stages (Shao et al., 2021). In the present study, Fusobacteriota and Proteobacteri were higher bacterial phyla in the pre-weaning donkey foal feces than in post-weaning donkey foals, which is common in other animal species (Shao et al., 2021). Gharechahi et al. (2021) observed that the Firmicutes and Fibrobacterota were mainly enriched for genes related to the degradation of lignocellulosic polymers and the fermentation of degraded products into short chain volatile fatty acids (VFA). Firmicutes have the potential to degrade a wide range of carbohydrate polymers, which have also been reported in numerous ruminants such as moose (Svartström et al., 2017), camel (Gharechahi and Salekdeh, 2018), and cattle (Stewart et al., 2019). Fibrobacterota were particularly adapted to degrade glucan, cellulose, arabinan, xylan, and xyloglucan (Gharechahi et al., 2021). Spirochaetota also demonstrated a high ability for lignocellulose degradation (Svartström et al., 2017). Due to their ability to target cellulose-xyloglucan polysaccharide complexes through genes, Spirochaetota have been reported to play a significant role in the hemicellulose breakdown in termite hindgut microbiota (Tokuda et al., 2018; Liu N. et al., 2019). With respect to donkey foals in this study, the degradation of fibre probably enhanced the establishment of the Firmicutes, Spirochaetota and Fibrobacterota colonization, as donkey foals no longer consume breast milk after weaning, but mainly feed on plant-based feeds. In addition, the gut microbiota has been linked with donkey body measurements. With the donkey body measurements increasing, the bacterial Firmicutes, Spirochaetota and Fibrobacterota were also increased. This result may indicate that the Firmicutes, Spirochaetota and Fibrobacterota promoted energy acquisition and body fat accumulation in donkey foals as cellulose and hemicellulose decomposition capacity increased in donkey foals from pre-to post-weaning period.

At genus level, the abundance of *Fusobacterium* and *Escherichia*-*Shigella* in pre-weaning donkey foals were higher than in dur- and post-weaning groups. It has been reported that fecal microbiota of neonates is age and growth dependent and its composition is closely related to pre-weaned animal health (Song et al., 2019). The *Fusobacterium* and *Escherichia*-*Shigella* presumably contribute to immunity development in the gut due to their capability to consume colostral oligosaccharides (Liu Y. et al., 2019). *Escherichia*-*Shigella* have also formed symbiotic relationships with anaerobes that require mono- and disaccharides (Maltby et al., 2013). Furthermore, *Escherichia*-*Shigella* participate in oxygen scavenging to contribute to an anaerobic environment (Jones et al., 2011). These activities could explain why *Escherichia*-*Shigella* is so prevalent in the pre-weaning donkey foal gut. In addition, *Escherichia-shigella* genus are usually associated with animal diarrhea in other species, such as pig and calf (Chanter et al., 1986; Song et al., 2019). Therefore, the lower abundance of this genus in the dur- and post-weaning groups compared to pre-weaning donkey foals suggests that donkey foals after weaning may have less prevalence of diarrhea caused by these opportunistic pathogens.

Conversely, the proportion of *Rikenellaceae*_RC9_gut_group, norank_f_F082, *Treponema*, NK4A214_group, *Lachnospiraceae*_AC2044_group and *Streptococcus* in PreW donkey foals were higher in DurW and PostW than in PreW donkey foals. Cui et al. (2022) noted that the *Rikenellaceae*_RC9_gut_group may play an important role in the biosynthesis of VFAs and energy utilization due to it was positively correlated with VFA production. Yi et al. (2022) reported that the abundance of norank_f_F082 was positively corelated with the proportion of concentrate. Therefore, the enriched norank_f_F082 genera in dur- and post-weaning groups may play an important role in the digestion of plant-based solid feed containing a lot of non-structural carbohydrates. *Treponema* has been reported to be cellulolytic bacteria in previous study (Evans et al., 2011). The increasing abundance of *Treponema* in donkey foals from pre-weaning to post-weaning period may be attributed to their capability to degrade plant celluloses (Li et al., 2020). The NK4A214_group belongs to the *Ruminococcus* family (Yi et al., 2022), and the high abundance in dur- and post-weaning groups may be related to its ability to digest plant fiber, because diets after weaning feature more plant fiber. In addition, they also produce pili involved in the bacterium’s adhesion to cellulose (Yi et al., 2022). *Lachnospiraceae*_AC2044_group was usually correlated with the propionate, and they ferment glucose to produce lactic acid (Han et al., 2021). The introduction of plant-based diets in dur- and post-weaning donkey foals may provide a large amount of fermentable carbohydrates that favored the growth of *Lachnospiraceae*_AC2044_group. Moreover, the easy availability of glucans in the substrate after weaning in donkey foals presumably promoted the colonization of fast-growing *Streptococcus*. Recently, Gharechahi et al. (2021) demonstrated that a high proportion of *Streptococcus* is observed when animals are offered concentrate. In accordance with the changes in bacterial genus during weaning period, the bacterial *Rikenellaceae*_RC9_gut_group, norank_f_F082, *Treponema*, NK4A214_group, *Lachnospiraceae*_AC2044_group and *Streptococcus* were positively correlated with donkey body measurements. By observing the body size of donkey foals from pre-to post-weaning period, we speculated that the large body size of post-weaning donkey foals might be related to the high cellulolytic bacteria content in their intestinal microbiota. After weaning, a high input of plant material in donkey foal hindgut may be hydrolyzed by these bacteria to produce more energy for bodily processes, which increased the body size of donkey foals.

Until now, little studies explored the bacterial function of the fecal microbiome in donkey foals during weaning transition. In the current study, we used PICRUSt software to determine the potential functional capacity of fecal bacteria of donkey foals from pre-weaning to post-weaning period. From pre-weaning to post-weaning period in donkey foals, the amounts and types of carbohydrates in breast and plant-based solid feeds are different, as well as the fermentation capacity of microbiota. The bacterial functions related to microbial metabolism and steroid hormone biosynthesis were significantly enriched in the fecal microbiome in the pre-weaning donkey foals. While the microbial gene functions, including the fatty acid biosynthesis, biosynthesis of secondary metabolites, Valine, leucine, and isoleucine biosynthesis, phenylalanine tyrosine and tryptophan biosynthesis, peptidoglycan biosynthesis and biosynthesis of amino acids, were mainly enriched in the pre-weaning donkey foals, and were linked with the carbohydrate metabolism and amino acid biosynthesis. These results indicated that the potential functions of bacteria in donkey foal feces are synergistic with host functions. The microbiota resident in the pre-weaning donkey foals were more inclined to have functions in the microbial metabolism and breast milk utilization, while microbiota resident in the dur- and post-weaning donkeys were more inclined to have functions such as fatty acid biosynthesis, secondary metabolites biosynthesis and amino acid biosynthesis. However, further studies are still required to characterize the microbial functions of gut bacteria in donkey foals during weaning transition.

### 4.2. Fecal anaerobic fungi changes in donkey foals

Anaerobic fungi have been most extensively studied in ruminants, but they have been found in both domesticated and wild equine species (Hess et al., 2020). In the present study, Neocallimastigomycota was found to be the most predominant fungal phylum in donkey foal feces, which is consistent with previous studies in goats and heifers (Mao et al., 2016; Zhang et al., 2017). Neocallimastigomycota play an essential role in the degradation of fibrous plant materials in herbivores (Gruninger et al., 2014). At genus level, the *Caecomyces, unclassified_c__Neocallimastigomycetes, Piromyces, unclassified_o__Neocallimastigales* and *Orpinomyces* were the predominant fungi genera in donkey foal feces. The predominated *Caecomyces* genera has previously been reported to occur in the equine feces and pony caecum (Liggenstoffer et al., 2010; Hanafy et al., 2020), and they have also been cultured from equines (Edwards et al., 2020). Metabolic profiles have previously been investigated for equine and rumen strains of *Piromyces*, with equine *Piromyces* possessing higher fiber degradation capacity in comparison with rumen isolates (Julliand et al., 1998). The *Orpinomyces* are also powerful fiber degraders due to their highly effective plant degrading enzymes, which including cellulase and xylanase activities (Youssef et al., 2013). From pre-weaning to post weaning, the anaerobic fungi composition in donkey foal feces were unchanged dramatically. Only the relative abundance of unclassified_o__*Neocallimastigales* were gradually decreased, and the proportion of *Orpinomyces* were the highest in DurW groups. The anaerobic fungus *Orpinomyces* is capable of growth on a variety of lignocellulosic materials and has a large reservoir of carbohydrate active enzymes (Morrison et al., 2016). Weaning of donkey foals usually occur between 5 to 6 months of age, but the shifts from milk to plant-based feeds is gradual. In the DurW group, donkey foals will have started ingesting forage and concentrates before weaning, which will promote the maturation of the digestive tract in preparation for the eventual change to forage-based feeds. Therefore, the forage-based feed with large quantity of fiber and lignocellulose may increase the abundance of *Orpinomyces* in the DurW donkey foals. A positive correlation between fungus *Orpinomyces* and body measurements of donkey foals was observed in the present study. This result means that the *Orpinomyces* may be associated with body size in donkey foals. The anaerobic fungi have been reported to be the primary degraders of fiber in herbivores (Edwards et al., 2020). The higher abundance of *Orpinomyces* in post-weaning donkey foals may promoted the VFA production, which ultimately promoted the body measurements in donkey foals.

To data, rare study was performed on the potential functional abundance of anaerobic fungi within donkey foals. In this study, FUNGuild was applied to infer the metabolic functional variations in anaerobic fungi among pre-, dur-, and post-weaning donkey foal feces. FUNGuild is a tool for assigning fungi trait information based on matching to a taxonomic classification using the FUNGuild database (Xie et al., 2021). The results showed that there were no obvious differences for anaerobic fungi functional abundance in donkey foals from pre-weaning to post-weaning period. This result might indicate that the fungal colonization of the foal gut commences in pre-weaning period. However, the role of these anaerobic fungi in the pre-weaning donkey foal hindgut ecosystem is currently unclear. Further studies will be required to understand why there were anaerobic fungi in the donkey foal gut before weaning without plant materials.

### 4.3. Fecal archaea changes in donkey foals

Similarly to the rumen, the donkey hindgut also accommodates a number of archaea that work symbiotically with bacteria and anaerobic fungi to degrade and ferment the feeds ingested by the host (Edwards et al., 2020). Many studies have been focused on the bacterial and anaerobic fungi diversity in the hindgut of adult equine (Liu et al., 2020; Zhang et al., 2022a,c). However, only limited investigation is available on the archaeal composition in donkey foals. The sequences detected at archaeal phylum in our study clustered into the Euryarchaeota, which is in accordance with the previous study of Murrua et al. (2018) in horse. The current PCoA of the methanogen community structure showed that in donkey foal feces there was a significant gap between the communities before and post weaning, but no obvious differences were observed between DurW and PostW groups. This finding suggested that weaning stress had a significant influence on the composition of the donkey foal methanogenic archaeal community.

Until now, the initial colonization of methanogens in the donkey foals has not yet been studied. In ruminants, Skillman et al. (2004) reported that the *Methanobrevibacter* were detected in the rumen liquid of lambs at the age of 3 days. In the present study, the predominant archaeal genera in donkey foal feces before weaning were also *Methanobrevibacter*, thereby implying that the initial colonization and establishment of methanogens in donkey foals began prior to weaning. Before weaning, H_2_ could be produced during carbohydrate fermentation. Methanogens use H_2_ as a source of energy to convert CO_2_ or acetate to CH_4_ during methanogenesis (Wang et al., 2017). Guzman et al. (2015) noted that the *Proteobacteria*, *Ruminococcus flavefaciens*, or other species are thought to provide methanogens with the necessary H_2_ and electrons for methanogenesis during the early stages prior to forage ingestion. After weaning, the relative abundance of *Methanocorpusculum* in donkey foals increased remarkably. Besides the change of diet structure and components, weaning transition also involves both psychological and physiological stress as the donkey foals were no longer raised alongside the dams. Therefore, the weaning may be a major event responsible for reshaping of the gut archaea in donkey foals. In the feces of adult horses, Lwin and Matsui (2014) identified the *Methanomicrobiales*, represented by *Methanocorpusculum*, as the predominate order and the *Methanobrevibacteria* as the less common species. In addition, the *Methanocorpusculum* has also been reported in adult donkey feces (Liu et al., 2014). Both the *Methanobrevibacter* and the *Methanocorpusculum* are hydrogenotrophic methanogens, which can also use formate, but their preference of different ecological niches is evident. The *Methanocorpusculum* are demonstrated as endosymbionts of anaerobic ciliates (Murrua et al., 2018). The abundance of methanogens related to the *Methanocorpusculum* in the equus species may be associated with the symbiotic relationship with the hydrogen producing “equine” anaerobic fungi and ciliates (Liggenstoffer et al., 2010). After weaning, plant feed particles may provide anaerobic fungi and ciliates with an abundant ecological niche. As a result, the increase abundance of anaerobic fungi and ciliates might encourage the growth of *Methanocorpusculum*.

For the anaerobic fermentation processes, the CH_4_ is released by donkeys as a by-product of enteric food digestion to maintain hydrogen at low partial pressures (Murrua et al., 2018). However, information regarding the loss of feed energy for a host animal and the composition of methanogenic microbial community in donkeys is limited. In the present study, there was a negative correlation between donkey body measurements and Euryarchaeota, and the BH, BL, TG, TW, RH, RL, RW, and CB of donkey foals were negatively correlated with *Methanobrevibacter*. The lower abundance of archaea means less loss of feed energy, thus, the decrease of methanogens in post-weaning donkey foals may enhance the body measurements of donkey foals by the improvement of feed efficiency. But in this study, there was also a positive correlation between donkey body measurements and *Methanocorpusculum* abundance. The composition of the archaea in the hindgut of equine could depend on the hindgut capacity as a reflection of body size (Lwin and Matsui, 2014). More investigation is needed in the future to understand the diversity and abundance of methanogens in the donkey foals for reducing CH_4_ emission.

In summary, the present study provides integrative information on the global fecal microbiota of healthy donkey foals undergoing the weaning transition. From pre-weaning to post-weaning, the cessation of breastfeeding gradually and weaning onto plant-based feeds increased the microbial diversity and richness. The cellulolytic related bacteria including phylum Firmicutes, Spirochaetota, and Fibrobacterota and genus norank_f_F082, *Treponema*, NK4A214_group, *Lachnospiraceae*_AC2044_group and *Streptococcus* increased. Meanwhile, the functions related to the carbohydrate metabolism and amino acid biosynthesis were obviously enriched in the fecal microbiome in the dur- and post-weaning donkeys. In terms of anaerobic fungi and archaea, the present study provided the first direct evidence that the initial colonization and establishment of anaerobic fungi and archaea in donkey foals began prior to weaning. In addition, the changes in the composition of the anaerobic fungi and archaea occurred during the weaning period. Altogether, the current study contributes to a better understanding of the development of the microbiota community in donkey foals from the pre-weaning to the post-weaning period. Future research should focus on the interactions of the metabolome, microbiome, and the gut functional development, as well as the long-term impact of manipulation during the weaning period on donkey production.

## Data availability statement

The datasets presented in this study can be found in online repositories. The names of the repository/repositories and accession number(s) can be found at: https://www.ncbi.nlm.nih.gov/, PRJNA903673.

## Ethics statement

The animal study was reviewed and approved by the Animal Welfare Committee of Liaocheng University.

## Author contributions

ZZ contributed to the manuscript writing, editing, data generation and analysis, revision, and general ideas of the manuscript. ZZ, BH, XS, XW, TW, and YW contributed to all the sample collection, results discussion, and collection of literature. GL and CW contributed to the conceptualization of the study, study fund support, results discussion, and draft revision. All authors read and approved the manuscript.

## Funding

This study was funded by the Shandong Province Modern Agricultural Technology System Donkey Industrial Innovation Team (grant number SDAIT-27), Livestock and Poultry Breeding Industry Project of the Ministry of Agriculture and Rural Affairs (grant number 19211162), Shandong Rural Revitalization Science and Technology Innovation Action Plan (Key Technology Innovation and Demonstration of Integrated Development of Dong-E Black Donkey Industry, grant number 2021TZXD012), Open Project of Liaocheng University Animal Husbandry Discipline (grant number 319312101–14), Open Project of Shandong Collaborative Innovation Center for Donkey Industry Technology (grant number 3193308), Research on Donkey Pregnancy Improvement (grant number K20LC0901), and Liaocheng University Scientific Research Fund (grant number 318052025).

## Conflict of interest

The authors declare that the research was conducted in the absence of any commercial or financial relationships that could be construed as a potential conflict of interest.

## Publisher’s note

All claims expressed in this article are solely those of the authors and do not necessarily represent those of their affiliated organizations, or those of the publisher, the editors and the reviewers. Any product that may be evaluated in this article, or claim that may be made by its manufacturer, is not guaranteed or endorsed by the publisher.

## Footnotes

^1^ http://drive5.com/uparse/

^2^ https://sourceforge.net/projects/rdp-classifier/
